# A Simple and Cost-Effective Retractor for Transorbital Neurosurgery: Technical Note and Application in Lacrimal Keyhole Approaches

**DOI:** 10.3390/jcm15020482

**Published:** 2026-01-07

**Authors:** Luca Ferlendis, Arianna Fava, Thibault Passeri, Rosaria Abbritti, Sebastien Froelich

**Affiliations:** Department of Neurosurgery, Lariboisière Hospital, Assistance Publique—Hopitaux de Paris, University of Paris, 75010 Paris, France; arianna.fava@aphp.fr (A.F.); thibault.passeri@aphp.fr (T.P.); rosaria.abbritti@aphp.fr (R.A.); sebastien.froelich@aphp.fr (S.F.)

**Keywords:** transorbital, neuroendoscopy, ventral skull base, retractor, lacrimal keyhole, technical advancements

## Abstract

**Background**: Transorbital approaches (TOAs) provide minimally invasive access to anterior and middle cranial fossa lesions. However, orbital retraction remains a challenge, as narrow corridors limit maneuverability and excessive retraction increase complication risk. Conventional rigid or malleable retractors may obstruct the corridor or exert uneven pressure on delicate tissues. We present a handmade, semi-rigid plastic retractor as a low-cost, effective solution to optimize orbital retraction in TOAs. **Methods**: The retractor was fashioned from a cylindrical plastic drill bit container, cut into two semicircular pieces with rounded edges. Its application is described within the transorbital eyebrow lacrimal keyhole approach (TELKA). During the bony phase, one piece is placed on the orbital roof for periorbital retraction and protection, while a second may be positioned laterally to protect the temporalis muscle when required. Once adequate working space is achieved, the lateral retractor is removed and the medial one maintained throughout the procedure. Technical details are illustrated through representative clinical cases, supported by anatomical dissection and an operative video. **Results**: Across thirteen TELKA procedures, the semi-rigid retractor provided stable, low-intensity retraction with even pressure distribution, minimizing corridor obstruction and facilitating both microscopic and endoscopic maneuverability. No orbital or visual complications related to retraction were observed; periorbital structures were preserved, with no postoperative proptosis or aesthetic defects. **Conclusions**: This handmade, semi-rigid retractor is a safe, customizable, and reproducible tool that enhances surgical freedom while minimizing orbital morbidity in TOAs. It is particularly advantageous in keyhole procedures such as TELKA, representing a promising alternative to conventional retraction systems.

## 1. Introduction

Transorbital approaches (TOAs) have gained increasingly recognition in recent years as minimally invasive corridors to access a variety of skull base pathologies located within the anterior and middle cranial fossae. These approaches provide a ventral and superolateral trajectory, enabling access while avoiding cerebral retraction [1,2,3,4].

However, orbital content retraction remains a major technical challenge in TOAs. The orbital content may obscure the surgical field and excessive manipulation risks complications such as globe contusion, periorbital hematoma, visual disturbances, or cranial nerve injury [5,6]. Manual retraction with thin malleable instruments is commonly employed but provides limited stability and non-uniform pressure distribution [1,7].

Rigid cylindrical retractors have recently been proposed to improve stability and increase the surgical corridor [8]. Although they provide a stable working corridor, their rigidity may lead to instrument conflicts, reduced maneuverability, and potentially injury to fragile intraorbital structures.

Several retraction systems, including self-retaining devices, tubular access channels, and balloon-assisted retractors, have been developed for deep-seated and intraventricular lesions [7,9,10,11]. However, these tools are not specifically designed for the orbital environment and may be impractical or overly invasive when applied to TOAs. Furthermore, maintaining a stable operative corridor during bone drilling and dural exposure is particularly challenging due to the restricted anatomy, where safe globe retraction is generally limited to approximately 10–15 mm [8,12,13,14].

Herein, we present a novel, simple, and cost-effective semi-rigid handmade orbital retractor designed to enhance surgical freedom and reduce orbital morbidity during the transorbital endoscopic lacrimal keyhole approach (TELKA) [15,16].

## 2. Materials and Methods

**Figure 1:**
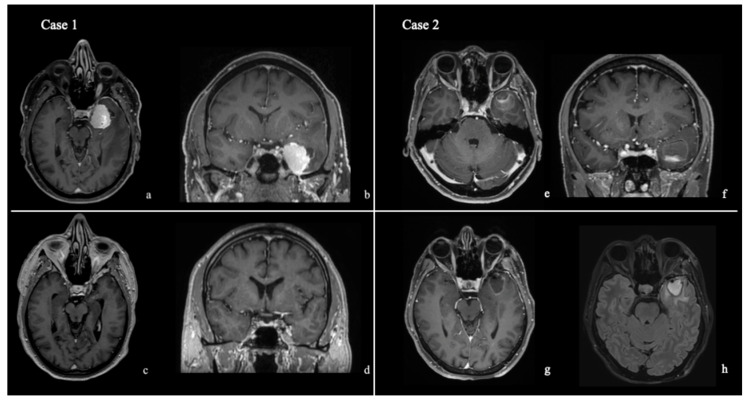
(**Case 1**) ref. [15]: A 64-year-old man presented with headache and anomia. (**a**,**b**) Preoperative contrast-enhanced T1-weighted MRI showing a left meningioma arising from the lateral wall of the cavernous sinus, associated with temporal lobe edema. Postoperative MRI (**c**,**d**) at 2-month follow-up demonstrating complete tumor removal (Simpson grade II). No postoperative visual, functional, or aesthetic deficits were observed. (**Case 2**): A 53-year-old woman presented with focal temporal seizures due to a temporal pole cavernoma. Surgical indication was established after documented rebleeding of the lesion. (**e**,**f**) Preoperative contrast-enhanced T1-weighted MRI in axial and coronal planes. (**g**,**h**) Postoperative MRI (contrast-enhanced T1-weighted and FLAIR sequences) showing complete lesion removal, with no evidence of orbital displacement or postoperative orbital edema following the TELKA. No postoperative visual or functional deficits were observed.

The retractor is routinely employed in our TELKA. The TELKA is selected for lesions involving the temporal pole, the lateral aspect of the cavernous sinus, and the mesial temporal lobe. To illustrate the surgical workflow and retractor application, representative cases are presented (Figure 1).

The entire technique, including retractor preparation, positioning, and intraoperative use, is demonstrated in the accompanying operative video (Video S1). In addition to the illustrative clinical case, an anatomical dissection was performed to qualitatively and quantitatively assess orbital retraction during the different bony phases of the TELKA.

A detailed step-by-step description of the technique is provided below.

### 2.1. Semi-Rigid Handmade Retractor Customization

**Figure 2:**
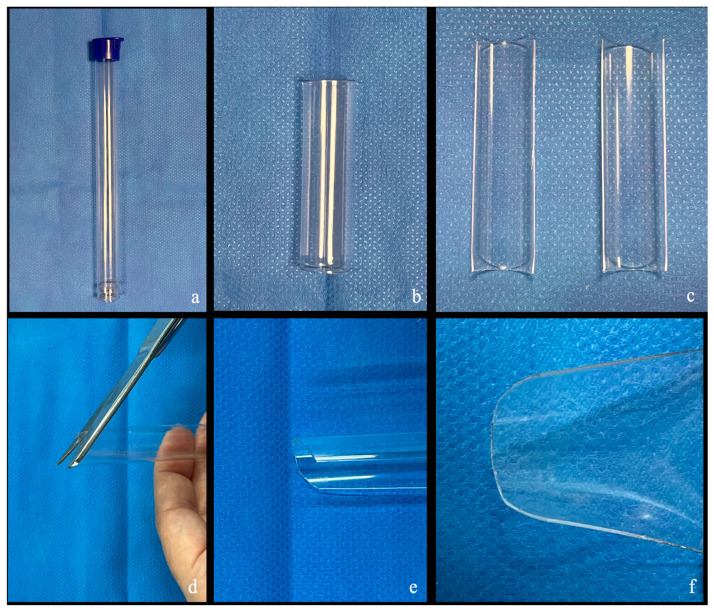
Step by step creation of the plastic orbital retractor. (**a**) A cylindrical container from a drill commonly used in neurosurgery is selected. (**b**,**c**) The tube is cut in half, obtaining two symmetrical parts. (**d**–**f**) The edges are rounded to prevent tissue damage and create two semi-rigid retractors.

At the beginning of surgery, a cylindrical drill bit container is selected. The plastic tube is cut longitudinally to obtain two symmetrical halves. Their length and width are refined according to the size of the corridor. The edges are rounded to create two semi-rigid, semicircular, hand-shaped retractors (Figure 2). The curvature can be further refined intraoperatively as needed.

### 2.2. Skin and Craniotomy

**Figure 3:**
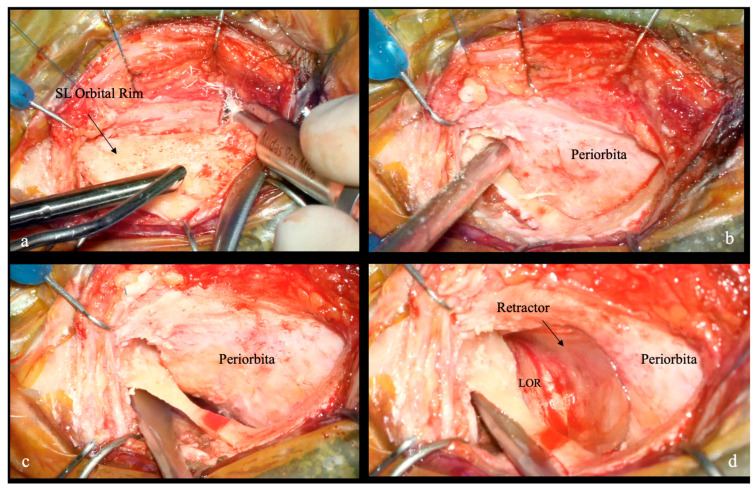
Intraoperative application of the plastic retractor during a left TELKA. (**a**) A crescent-shaped superolateral orbital rim craniotomy is performed, extending from the supraorbital foramen to the frontozygomatic suture. (**b**,**c**) Exposure of the superolateral orbital wall and the periorbita, which is detached from the orbital wall up to the lateral aspect of the superior orbital fissure (SOF). (**d**) The periorbita is then safely retracted using the previously created retractor, allowing drilling of the orbital roof and greater sphenoid wing while avoiding orbital manipulation. Abbreviations: lateral orbital roof (LOR).

The patient is positioned supine, the head is fixed in a Mayfield head holder, rotated approximately 10° contralaterally in slight extension to facilitate the natural corridor toward the greater sphenoid wing (GSW). Neuronavigation and intraoperative cranial nerves monitoring are used. The procedure is performed using an operating microscope (OM). A skin incision is made within the lateral third of the eyebrow. After incising the orbital muscle and periosteum, the superolateral orbital rim is exposed subperiosteally. The periorbita is carefully elevated to expose the orbital roof. A lachrymal keyhole craniotomy is performed by removing the tip of the superolateral orbital rim in a semilunar fashion, from the supraorbital foramen to the frontozygomatic suture. The lateral orbital roof, and orbital aspect of the zygoma and GSW, are exposed. The retractor is then modeled and anchored to the orbital roof to gently retract the periorbita (Figure 3).

### 2.3. Bony Phase

**Figure 4:**
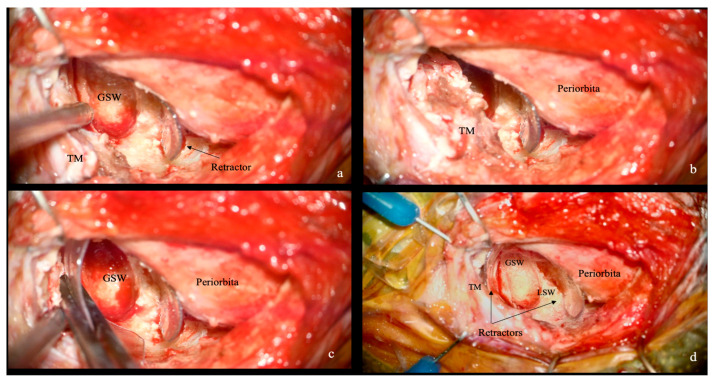
Intraoperative bony work and application. (**a**) The orbital contents are protected with the retractor while the orbital wall and greater sphenoid wing (GSW) are drilled laterally, exposing the deep periosteal layer of the temporalis muscle (TM). (**b**,**c**) Once the TM is exposed and enters the surgical field, a second plastic retractor can be used, as shown in (**c**), to maintain consistent retraction. (**d**) The two mirrored retractors expose the GSW and the lesser sphenoid wing (LSW).

The inner margin of the edge of the craniotomy is drilled to flare the opening toward the orbit and to expand the microscopic field of view. The orbital portion of the zygoma is drilled to expose the deep periosteal layer of the temporalis muscle (TM) and the GSW is drilled exposing the orbito-temporal periosteal fold (OTPF) and the temporal fossa dura. A second small retractor may be used if necessary to retract the TM to enhance exposure and protect the temporalis fascia (Figure 4).

### 2.4. Extradural and Intradural Phases

**Figure 5:**
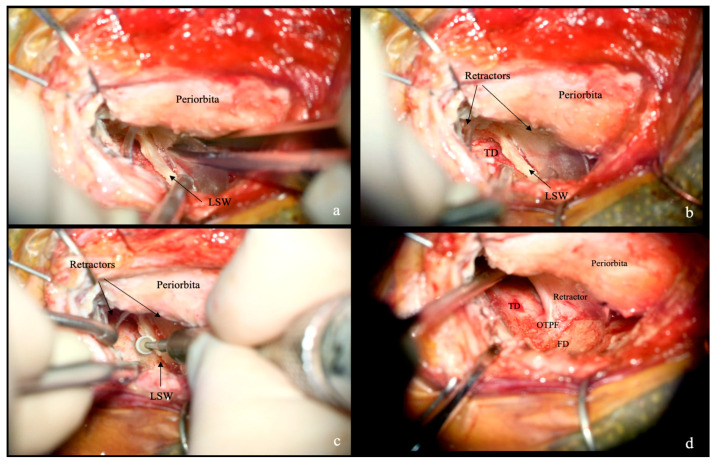
Intraoperative intracranial extradural phases of the approach. (**a**) The retractors are positioned to expose the lesser sphenoid wing (LSW) and the temporal dura (TD). (**b**) Due to the semi-rigid nature of the material, the retractors remain securely anchored to the bone while allowing movement by the surgical instruments, ensuring continuous protection of the neurovascular structures. In this manner (**c**,**d**), the LSW can be safely drilled until the TD, the frontal dura (FD), and the orbito-temporal periosteal fold (OTPF) are fully exposed.

After drilling the GSW and exposing the temporal dura, the retractors are repositioned to allow deeper work while protecting the periorbita medially and the temporal dura laterally. The lesser sphenoid wing (LSW) is drilled to optimize the visualization of the SOF and the orbito-temporal periosteal fold (OTPF) (Figure 5).

**Figure 6:**
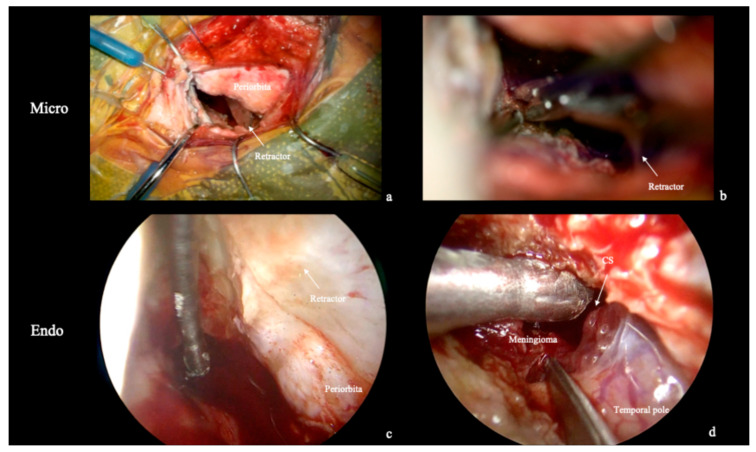
Extradural and intradural phase. (**a**) During both the microscopic and endoscopic phases of the surgery (Illustrative Case 1), the retractor remains in place to protect periorbita during instruments insertion and manipulation. (**b**) Microscopic Interdural peeling; (**c**,**d**) The retractor shields the periorbita while a 30° endoscope is used with the Endoscopic Chopstick technique to improve maneuverability during meningioma removal.

Once adequate bone exposure is obtained, the lateral retractor is removed, while the medial retractor remains in place throughout the procedure. The OM is used during the epidural and initial intradural phases; however, when visualization becomes insufficient during deeper intracranial steps, the procedure is continued using an endoscope. The Endoscopic Chopstick Technique [17], with angled scopes, is applied to improve maneuverability in the narrow surgical corridor (Figure 6). During these phases, the medial retractor remains in position to shield the intraorbital structures from instrument insertion and manipulation during both microscopic and endoscopic phases.

Closure is performed in a multilayer fashion as previously described [15]. Abdominal fat is used to fill the dead space, and the skin is closed with an intradermal suture.

## 3. Results

The handmade semi-rigid retractor was successfully used in thirteen TELKA procedures, including three lateral cavernous sinus meningiomas, one temporal pole meningioma, one temporal pole cavernoma, one trigeminal schwannoma, one spheno-orbital meningioma, and six transulcal selective amygdalohippocampectomies.

**Figure 7:**
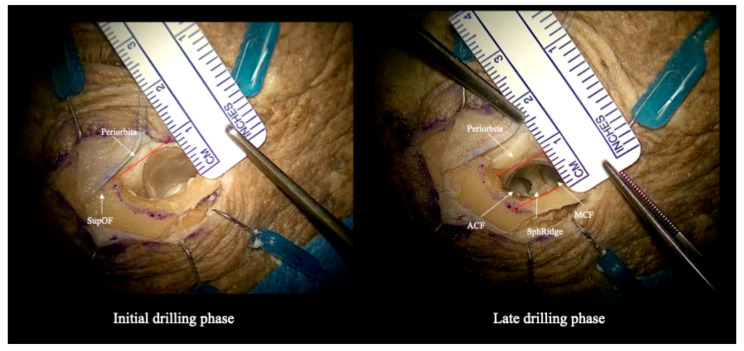
Anatomical dissection of a right TELKA. In the initial bony phase (**left**) the retraction is approximately 9 mm, while in the late drilling phase, gaining space, orbital medial retraction is slightly diminished to 7 mm on the (**right**). Red lines indicate distances measured in millimeters, perpendicular to the ruler visible in the images. Abbreviations: ACF: anterior cranial fossa; MCF: middle cranial fossa; SphRidge: sphenoidal ridge; SupOF: Superior orbital fissure.

During the initial bony phase (Figure 3 and Figure 4), when the superolateral orbital roof is still intact, the working corridor is more restricted and orbital retraction is maximal. With progressive drilling of the orbital roof and lateral orbital wall, the available space increases and the need for medial orbital retraction progressively decreases. As demonstrated by the anatomical specimen dissection (Figure 7), orbital medial displacement decreased from approximately 9 mm in the early phase to about 7 mm in the late drilling phase. In this phase, the retractor provided stable, low-intensity periorbital retraction, without interfering with instrument maneuverability, while effectively protecting the orbital contents.

During the deeper extradural phases, dynamic repositioning of the retractor avoided prolonged static pressure on the orbital structures. Once drilling of the lesser sphenoid wing was safely completed, its slim, low-profile design facilitated visualization of the dural structures while minimizing the need for orbital retraction (Figure 5).

During the intradural phase (Figure 6), orbital retraction requirements were reduced compared with the bony and extradural phases, and the retractor mainly functioned as a protective barrier, with retraction being predominantly dynamic and related to instrument insertion.

**Figure 8:**
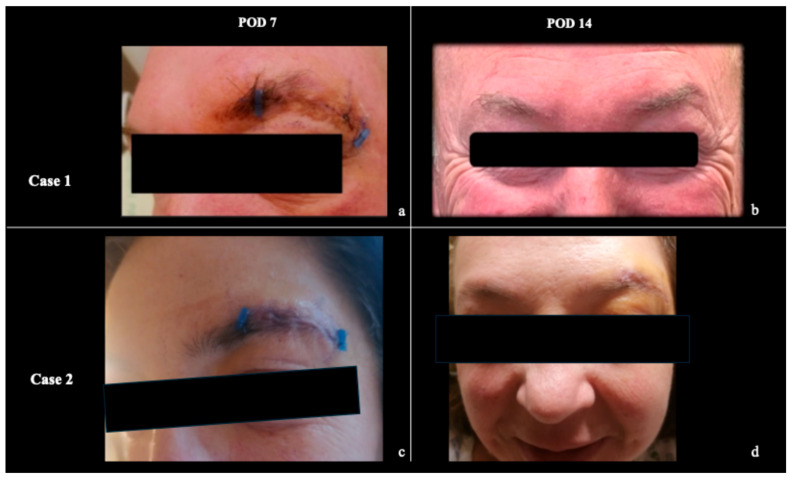
Postoperative outcomes of (**a**,**b**) **Case 1** and (**c**,**d**) **Case 2**. Sutures were removed at POD 7. At 14 days postoperatively, no aesthetic, functional, visual, or cranial nerve deficits were observed.

Clinically, no intraoperative complications related to orbital manipulation were observed. Intraoperative assessment confirmed preservation of the periorbital structures, and postoperative neurological and ophthalmological evaluations revealed no visual deficits across all 13 cases. Specifically, no cerebrospinal fluid leakage, oculomotor disturbances, ptosis, or clinically evident proptosis or enophthalmos were detected postoperatively (Figure 1). Cosmetic and functional outcomes were consistently favorable in all patients (Figure 8).

## 4. Discussion

Orbital retraction continues to pose a major challenge in TOAs. Safe periorbital preservation must be balanced with adequate surgical freedom and instrument maneuverability.

Depending on lesion location, TOAs often require medial or inferomedial displacement of the orbital contents. Recent quantitative investigations indicate thresholds for safe retraction, demonstrating that medial displacement exceeding 15 mm may critically increase intraocular pressure [13,14].

Malleable metallic retractors are commonly used to mitigate complications, but in TOAs performed without orbitotomy they can obstruct the corridor needed for an endoscope and two instruments [1,2,3,4,5]. Moreover, their manipulation often requires a second operator, further reducing the available workspace [6,7].

Rigid retraction systems have also been proposed as an alternative. Karimzada et al. [8] recently evaluated rigid tubular retractors in cadaveric TOA models and found that they provide more uniform circumferential pressure around the globe compared with soft retraction systems, reducing focal compression risk. However, their rigidity makes intraoperative repositioning difficult and often leads to conflicts with instruments, especially during bony drilling. In addition, the applicability of cadaveric findings is limited by postmortem desiccation of the globe [18], which affects compliance and reduces the reliability of surgical freedom and pressure assessments [10].

In contrast, the handmade semi-rigid retractor described here offers several advantages. Fashioned intraoperatively by halving a standard plastic drill bit container and trimming it to match the orbital roof depth, it provides a simple, customizable, and cost-effective solution. Its slim, low-profile design minimizes obstruction within keyhole corridors, improving surgical freedom. It is intended to provide stable protection of the intraorbital contents during the insertion and manipulation of instruments.

**Figure 9:**
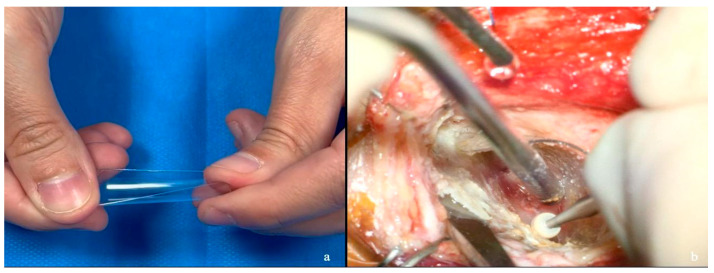
This image illustrates the flexibility and versatility of the handmade plastic retractor (**a**). In (**b**), its properties allow it to be dynamically adjusted and mobilized by the surgeon’s instruments.

The semicircular shape enables stable anchoring to the orbital roof while gently retracting periorbital tissues, facilitating uniform pressure distribution and minimizing focal stress. Its semi-rigid consistency provides steady, low-intensity retraction without focal pressure points associated with rigid devices [19]. Rounded edges enhance safety by reducing the risk of periorbital injury and permitting smooth repositioning. The lightweight device is easily displaced by instruments without creating mechanical conflicts, enhancing maneuverability (Figure 2 and Figure 9). Importantly, the retractor is continuously and dynamically repositioned during the procedure, avoiding prolonged static pressure on the orbital contents and adhering to the principle of dynamic “brain” retraction in skull base surgery. Compared with conventional malleable metallic retractors and rigid tubular systems, the proposed semi-rigid plastic retractor occupies an intermediate role, providing stable, low-profile orbital protection while remaining dynamically adaptable to instrument movement.

**Figure 10:**
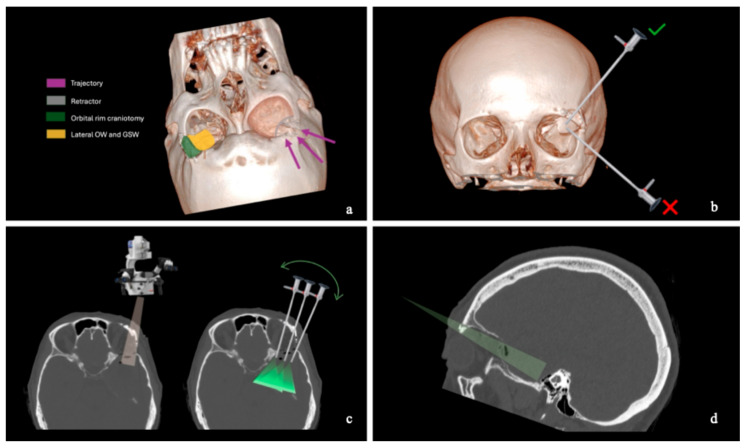
Illustrative representation of the transorbital surgical corridor with retractor application, based on a 3D reconstruction of the postoperative CT scan. (**a**) After the crescent-shaped superolateral orbital rim craniotomy, the lateral orbital wall (OW) and the greater sphenoid wing (GSW) are drilled. The purple arrows indicate the surgical trajectory. (**b**) In accordance with keyhole principles, the surgeon is positioned at the head of the patient. The green check mark indicates the endoscope position in the TELKA, whereas the red cross indicates the endoscope position in standard TOAs. (**c**) The orange and green light cones represent microscopic and endoscopic fields of view, respectively. The green arrow indicates the increased range of motion along the axial plane achievable with the endoscope, allowing deeper visualization compared with the operating microscope. (**d**) The green cone represents the surgical corridor along the sagittal plane. Overall, the orbital rim craniotomy improves the angle of attack along both the lateromedial and superoinferior axes. When the operating microscope does not provide sufficient visualization, the endoscope is employed to enhance visibility during the deeper phases of the procedure.

When combined with a superolateral orbitotomy in the TELKA, the increase in surgical freedom and angle of attack is primarily achieved by the bony work rather than by the retractor itself. As previously described by our group [15,16], the TELKA integrates principles of the supraorbital keyhole and endoscopic transorbital routes. An eyebrow incision is made and the surgeon is positioned at the head of the patient, rather than TOAs described by other authors [3,4,20,21], which typically use an upper eyelid incision and a different surgeon positioning facing the patient. Removal of the superolateral orbital rim improves the line of sight and widens the operative corridor along both superoinferior and lateromedial axes (Figure 10). This modification of the corridor substantially reduces the need for intrinsic globe retraction.

Nevertheless, particularly during the initial phase of the approach, a degree of orbital retraction remains necessary. In this context, the retractor assists in minimizing orbital manipulation while maintaining a stable surgical field. During the deeper phases of the procedure, once the bony corridor has been sufficiently enlarged, the semi-rigid retractor primarily serves a protective role, shielding the periorbital contents while preserving full maneuverability during both microscopic and endoscopic phases.

During extradural drilling, using the OM avoids endoscope-related crowding and eliminates the need for a second operator dedicated to retraction, thereby enhancing surgeon autonomy and workflow efficiency. A mirrored second retractor may also be used to protect the temporalis muscle, providing dynamic “tubular-like” retraction without the limitations of rigid tubes.

Across the reported surgical experience, orbital retraction was greatest during the initial bony phase and progressively decreased as drilling of the orbital roof and lateral orbital wall enlarged the corridor, a finding supported by anatomical dissection. Importantly, this strategy maintained orbital displacement within the accepted 15 mm safety threshold, potentially reducing the risk of intraocular pressure elevation and associated complications. No periorbital injuries or intraorbital complications were observed. The combined use of the retractor and the lacrimal keyhole approach reduced the need for orbital manipulation, contributing to lower rates of postoperative oedema and proptosis. Reconstruction of the orbital rim allowed satisfactory immediate aesthetic outcomes, while the eyebrow incision was associated with rapid healing and favorable cosmetic results.

To our knowledge, this represents the first technical report describing a low-cost, handmade semi-rigid retractor specifically adapted for TOAs and integrated into a reproducible keyhole workflow.

### Limitations

As a technical note, this study has inherent limitations. The handmade retractor lacks prospective validation or quantitative biomechanical assessment, such as pressure distribution analysis or intraocular pressure monitoring. Although the device was applied in multiple cases, the study was not designed as a comparative or statistical analysis. The findings are based on a single-center experience and are specific to the TELKA, applied on a specific approach (TELKA), which may limit generalizability to fully endoscopic transorbital techniques. While we believe that the proposed retractor may be applicable to a broad range of transorbital approaches, no direct comparison with conventional retraction systems or formal statistical evaluation of complication rates was performed. Further anatomical, biomechanical, and comparative clinical studies are warranted to validate these preliminary observations.

## 5. Conclusions

This handmade semi-rigid retractor offers a simple, cost-effective, and customizable adjunct to enhance surgical freedom while minimizing orbital morbidity in TOAs. Its ergonomic design allows stable, uniform distributed retraction with minimal corridor obstruction, which is particularly advantageous in keyhole procedures such as TELKA. Although further biomechanical validation and larger clinical studies are necessary, this device represents a promising alternative to conventional retraction systems, potentially improving safety and efficacy in transorbital skull base surgery.

## Data Availability

The original contributions presented in the study are included in the article/Supplementary Material, further inquiries can be directed to the corresponding author.

## Supplementary Materials

The following supporting information can be downloaded at: https://www.mdpi.com/article/10.3390/jcm15020482/s1, Video S1: Application of the Handmade Semi-Rigid Retractor During the TELKA.

## Abbreviations

The following abbreviations are used in this manuscript:

TOAs	Transorbital approaches
TELKA	transorbital endoscopic lacrimal keyhole approach
TM	Temporalis muscle
GSW	Great sphenoidal wing
OTPF	Orbito-temporal periosteal fold
LSW	Lesser sphenoidal wing
MRI	Magnetic resonance imaging
SOF	Supraorbital fissure
LOR	Lateral orbital roof
CT	Computed tomography

## References

[1] Jeon C., Hong C.-K., Woo K.I., Hong S.D., Nam D.-H., Lee J.-I., Choi J.W., Seol H.J., Kong D.-S. (2019). Endoscopic Transorbital Surgery for Meckel’s Cave and Middle Cranial Fossa Tumors: Surgical Technique and Early Results. J. Neurosurg..

[2] Kong D.-S., Kim Y.H., Hong C.-K. (2021). Optimal Indications and Limitations of Endoscopic Transorbital Superior Eyelid Surgery for Spheno-Orbital Meningiomas. J. Neurosurg..

[3] Di Somma A., Andaluz N., Cavallo L.M., de Notaris M., Dallan I., Solari D., Zimmer L.A., Keller J.T., Zuccarello M., Prats-Galino A. (2018). Endoscopic Transorbital Superior Eyelid Approach: Anatomical Study from a Neurosurgical Perspective. J. Neurosurg..

[4] Dallan I., Castelnuovo P., Locatelli D., Turri-Zanoni M., AlQahtani A., Battaglia P., Hirt B., Sellari-Franceschini S. (2015). Multiportal Combined Transorbital Transnasal Endoscopic Approach for the Management of Selected Skull Base Lesions: Preliminary Experience. World Neurosurg..

[5] De Rosa A., Pineda J., Cavallo L.M., Di Somma A., Romano A., Topczewski T.E., Somma T., Solari D., Enseñat J., Cappabianca P. (2019). Endoscopic Endo- and Extra-Orbital Corridors for Spheno-Orbital Region: Anatomic Study with Illustrative Case. Acta Neurochir..

[6] Vural A., Carobbio A.L.C., Ferrari M., Rampinelli V., Schreiber A., Mattavelli D., Doglietto F., Buffoli B., Rodella L.F., Taboni S. (2021). Transorbital Endoscopic Approaches to the Skull Base: A Systematic Literature Review and Anatomical Description. Neurosurg. Rev..

[7] Greenberg I.M. (1981). Self-Retaining Retractor and Handrest System for Neurosurgery. Neurosurgery.

[8] Karımzada G., Evleksiz Karımzada D., Erol G., Gülsuna B., Kuzucu P., Güngör A., Kutlay A.M., Şahin M.M., Çeltikçi E. (2024). Transorbital Neuroendoscopic Surgery for Treatment of Sphenoid Wing Meningiomas Extending to the Cavernous Sinus: Clinical Implications and a Technical Illustration. Neurosurg. Focus.

[9] Bander E.D., Jones S.H., Kovanlikaya I., Schwartz T.H. (2016). Utility of Tubular Retractors to Minimize Surgical Brain Injury in the Removal of Deep Intraparenchymal Lesions: A Quantitative Analysis of FLAIR Hyperintensity and Apparent Diffusion Coefficient Maps. J. Neurosurg..

[10] Zammar S.G., Cappelli J., Zacharia B.E. (2019). Utility of Tubular Retractors Augmented with Intraoperative Ultrasound in the Resection of Deep-Seated Brain Lesions: Technical Note. Cureus.

[11] Kashimura H., Ogasawara K., Kubo Y., Kakino S., Sasoh M., Takahashi H., Suzuki K., Ogawa A. (2008). Brain Retraction Technique Using Gelatin Sponge in the Subtemporal Approach. Neurol. Med. Chir..

[12] Bly R.A., Ramakrishna R., Ferreira M., Moe K.S. (2014). Lateral Transorbital Neuroendoscopic Approach to the Lateral Cavernous Sinus. J. Neurol. Surg. B Skull Base.

[13] Piper K., Saez-Alegre M., George Z., Srivastava A., Felbaum D.R., Jean W.C. (2024). Transorbital Surgical Corridor: An Anatomic Analysis of Ocular Globe Retraction and the Associated Exposure for the Transpalpebral Orbital Rim Preserving Endoscopic Orbitotomy (TORPEDO) Approach. Oper. Neurosurg..

[14] Kim W., Moon J.H., Kim E.H., Hong C.-K., Han J., Hong J.B. (2021). Optimization of Orbital Retraction during Endoscopic Transorbital Approach via Quantitative Measurement of the Intraocular Pressure-[SevEN 006]. BMC Ophthalmol..

[15] Fava A., Jiang T., Vu T.H., Abbritti R., Froelich S. (2025). Transorbital Eyebrow Lacrimal Keyhole Approach for Resection of a Meningioma of the Lateral Wall of the Cavernous Sinus. Neurosurg. Focus Video.

[16] Matano F., Passeri T., Abbritti R., Camara B., Mastantuoni C., Noya C., Giammattei L., Devaux B., Mandonnet E., Froelich S. (2022). Eyebrow Incision with a Crescent-Shaped Orbital Rim Craniotomy for Microscopic and Endoscopic Transorbital Approach to the Anterior and Middle Cranial Fossa: A Cadaveric Study and Case Presentation. Brain Spine.

[17] Ferlendis L., Watanabe N., Fava A., Jiang T., Passeri T., Froelich S. (2025). Mononostril Endoscopic Endonasal Chopstick Technique for Low Petroclival Meningioma with Sphenoidal Sinus Cranialization and Rostral Mucosal Closure. Oper. Neurosurg..

[18] Noiphithak R., Yanez-Siller J.C., Revuelta Barbero J.M., Otto B.A., Carrau R.L., Prevedello D.M. (2019). Comparative Analysis Between Lateral Orbital Rim Preservation and Osteotomy for Transorbital Endoscopic Approaches to the Cavernous Sinus: An Anatomic Study. Oper. Neurosurg..

[19] Kelly P.J. (1989). Future Perspectives in Stereotactic Neurosurgery: Stereotactic Microsurgical Removal of Deep Brain Tumors. J. Neurosurg. Sci..

[20] Castelnuovo P., Turri-Zanoni M., Battaglia P., Locatelli D., Dallan I. (2015). Endoscopic Endonasal Management of Orbital Pathologies. Neurosurg. Clin. N. Am..

[21] Locatelli D., Restelli F., Alfiero T., Campione A., Pozzi F., Balbi S., Arosio A., Castelnuovo P. (2022). The Role of the Transorbital Superior Eyelid Approach in the Management of Selected Spheno-Orbital Meningiomas: In-Depth Analysis of Indications, Technique, and Outcomes from the Study of a Cohort of 35 Patients. J. Neurol. Surg. B Skull Base.

